# Anterior Vertebral Body Tethering (AVBT) in the Treatment of Adolescent Idiopathic Scoliosis: A Retrospective Study

**DOI:** 10.3390/jcm13247786

**Published:** 2024-12-20

**Authors:** Massimo Balsano, Andrea Vacchiano, Mauro Spina, Federico Lodi, Pietro Criveller, Fabio Zoccatelli, Alberto Corbolini, Antonio Gioele Colombini, Alessio Campisi, Riccardo Giovannetti, Maurizio Valentino Infante

**Affiliations:** 1 Regional Spinal Department, University and Hospital Trust, 37126 Verona, Italy; massimo.balsano@gmail.com (M.B.); andrea.vacchiano@hotmail.it (A.V.); 2 Orthopedics and Traumatology Department, University and Hospital Trust, 37126 Verona, Italy; lodifede@gmail.com (F.L.); pietri.criveller@gmail.com (P.C.); zoccatelli.fabio@gmail.com (F.Z.); alberto.corbolini@gmail.com (A.C.); antoniocolombini92@gmail.com (A.G.C.); 3 Thoracic Surgery Department, University and Hospital Trust—Ospedale Borgo Trento, 37126 Verona, Italy; alessio.campisi@aovr.veneto.it (A.C.); riccardo.giovannetti@aovr.veneto.it (R.G.); maurizio.infante@aovr.veneto.it (M.V.I.)

**Keywords:** anterior vertebral body tethering, AVBT, adolescent idiopathic scoliosis, fusion less

## Abstract

**Background/Objectives**: Anterior Vertebral Body Tethering (AVBT) is a relatively novel minimally invasive surgical technique for the treatment of adolescent idiopathic scoliosis (AIS) that enables deformity correction of the spine diminishing vertebral motion reduction caused by the standard posterior spinal fusion approach. This paper reports the introduction of a new technical variant of AVBT, with the aim of evaluating its effectiveness on the correction of both axial and coronal spinal deformity. **Methods**: A single-centre single-surgeon retrospective cohort study was conducted. AVBTs were performed between 2020 and 2024. Radiographical values, surgical details, and complications of 67 patients affected by AIS were compared before surgery, immediately after surgery, and at the most recent follow-up. **Results**: Postoperative results have revealed a statistically significant coronal curve correction of 29.85% in the main thoracic (MT) curves (from mean preoperative width of 54.81 ± 11.86° to 38.45 ± 10.19°) and of 26.93% in the thoracolumbar (TL/L) curves (from 35.15 ± 11.83° to 25.69 ± 10.50°) in line with that obtained by the standard technique. Coronal correction at the most recent follow-up was maintained. Postoperative axial rotation reduction was found to be statistically significant in the main thoracic (MT) curves (from mean Nash-Moe value of 1.84 ± 0.71 to 1.36 ± 0.73), with a further decrease at the most recent follow-up compared with preoperative values. Improvement in other radiographical measures did not reach statistical significance and the complication rate was comparable to the standard technique. **Conclusions**: The extent of coronal correction in patients treated with the proposed modified AVBT technique is satisfactory and in line with results from studies testing the standard AVBT technique. The findings of this study seem to suggest that this technical variant of AVBT is effective in the correction of both axial and coronal deformity, with a surgical complication rate comparable to the standard technique.

## 1. Introduction

Adolescent idiopathic scoliosis (AIS) is a complex tri-planar deformity of the spine characterized by altered Thoracic Kyphosis, lateral coronal deviation and axial rotation of vertebral bodies. It is the most common form of pediatric scoliosis, accounting for nearly 80% of cases.

Treatment of AIS depends on skeletal maturity, coronal curvature magnitude and curve progression speed and includes conservative management and observation, bracing and surgery. Surgical treatment is generally advised for patients with a coronal curvature of 45 Cobb degrees and over as these curves are more likely to progress, either with or without bracing, even after skeletal maturity [1,2].

Therefore, surgical intervention is recommended for curves of more than 50° Cobb in patients who have completed skeletal growth and for curves of more than 45° Cobb in children who are rapidly growing, since such curves are likely to progress before skeletal maturity [3].

The gold standard of AIS surgical treatment is posterior spinal fusion (PSF), which has proven to be effective in spinal deformity correction. Nevertheless, this surgical procedure leads to a permanent reduction in spinal motion and to an increased incidence of developing hardware complications, spondyloarthritis and degenerative disc disease above and below the levels where fusion surgery was performed (adjacent segment disease) [4].

For this reason, several surgical options have been investigated with the aim of correcting spinal deformity in skeletally immature patients by exploiting the spinal growth potential while preserving the spinal motion. One of these techniques is Anterior Vertebral Body Tethering (AVBT), a relatively new minimally invasive procedure possibly based on the Hueter-Volkmann law: by implanting an anterolateral flexible tether between adjacent vertebral bodies, a compressive force is produced, providing instant correction and growth modulation of the spine. Although AVBT techniques described in the literature enable the correction of vertebral rotation in the coronal plane, only a few studies have focused on evaluating the correction in the axial plane [5,6].

We adopted a new technical variant of AVBT that includes the hemi-discectomy at the apex of the main thoracic curve and the proximo-distal tensioning of the polyethylene terephthalate (PET) cord. The purpose of this study is to evaluate its effectiveness on addressing both axial and coronal deformity.

## 2. Materials and Methods

A single-centre single-surgeon retrospective cohort study of 67 patients affected by AIS who underwent AVBT was conducted. Surgeries were performed via video-assisted thoracoscopic surgery by a single senior spine surgeon at the Regional Spinal Department of the University and Hospital Trust of Verona (Italy) between 2020 and 2024.

Demographic data included sex and age at the time of surgical procedure. Curve type was assessed by Lenke classification. Skeletal maturity was determined by using Risser staging and Sanders Maturity Scale (SMS). Radiographic measurements were performed by five residents using Surgimap (Nemaris, Inc., New York, NY, USA) computer software, version number 2.3.2.1. Coronal parameters included Cobb Angles of main thoracic curves (MT), proximal thoracic curves (PT) and thoracolumbar/lumbar curves (TL/L), Clavicle Angle, Coronal Balance, and Trunk Shift (TS). Sagittal parameters included Thoracic Kyphosis (TK), Sagittal Balance, Lumbar Lordosis (LL), Thoracic Height, and Trunk Height. Axial parameters included Nash-Moe index of apex vertebra rotation of major structural MT and TL/L curves. Surgical data included the number and location of tethered vertebrae, operative time, complications and length of stay in hospital after surgery. Outpatient evaluations were performed preoperatively, immediately after surgery and at the most recent follow-up by four senior spine surgeons.

### Surgical Technique

**Figure 1:**
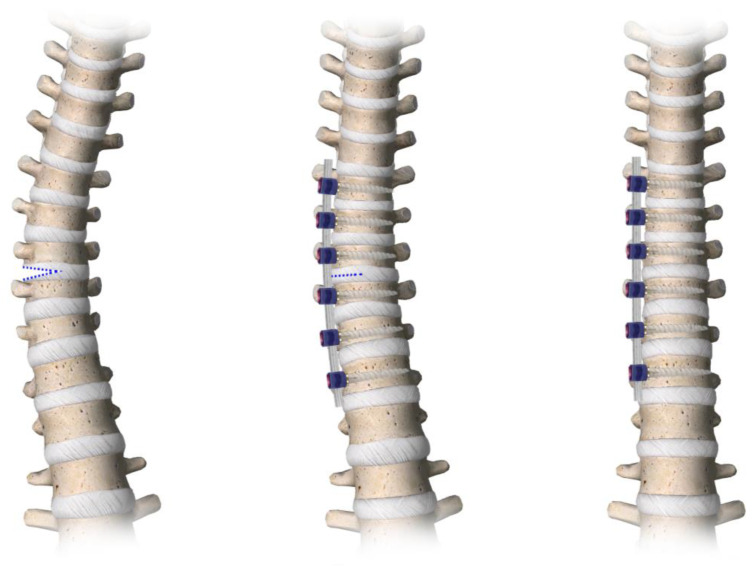
Graphical representation of the modified AVBT technique.

Surgery is performed in a minimally invasive way, placing the screws and the flexible polyethylene terephthalate (PET) cord under direct thoracoscopy visualization according to the modified technique described below and represented in Figure 1.

The patient is placed in lateral decubitus on the operating table with the convex side of the curve facing upwards and is secured by elastic bands around the hips and the lower limbs. Bony prominences are protected with soft pads. Single-lung ventilation is obtained with a double-lumen endotracheal tube and the lung at convex side of the MT curve is deflated. The position of vertebral bodies and the placement of thoracoscopic portals are identified through postero-anterior and lateral images of the spine obtained with C-arm fluoroscopy and the use of a straight metal marker and are then drawn on the overlaying skin. One thoracotomy access and two or three thoracoscopic portals are used: thoracotomy is performed at the apex of the curve, while thoracoscopic portals are placed along the midaxillary line. The parietal pleura is incised longitudinally anterior to the rib heads and the segmental vessels are cauterized. Under thoracoscopic visualization, hemi-discectomy at the apex of the curve is performed to increase its flexibility. The hemi-discectomy is first performed by opening the annulus on the convex side using a blade scalpel; subsequently, the removal of half of the nucleus pulposus on the convex side is carried out. At this point, a 3-prong staple is positioned on the anterior aspect of the vertebral body, adjacent to the rib head, and the screw hole is tapped under C-arm fluoroscopy checking. A probe is advanced across the hole and passed through the contralateral vertebral body cortex to determine the optimal length, and then one hydroxyapatite-coated screw is placed into the vertebra. In this phase, it is important to place the screw in a more ventral or dorsal portion of the vertebral body to allow de-rotation of the axial curve using tower devices secured to the screw heads. This procedure is repeated for each vertebra that needs to be instrumented. Hemi-discectomy is also performed at the apex of the curve (if it coincides with a disc) or, at most, at the two-disc levels adjacent to the apex of the curve (if it coincides with a vertebra) to allow for better flexibility and correction, by making an incision in the annulus on the convex side. Once all the screws are correctly placed, PET cord is introduced within every screw head. Coronal correction is achieved by forcing the curve with external maneuvers and de-rotation is applied by means of tower devices secured to the screw heads. Tether cord is then tensioned in the proximo-distal direction with the external tensioning device, applying major tension on the apex of the curve. The excess cord is cut 2 cm away from the cranial and caudal screws. At the end of the procedure, a thoracic drain is inserted through one of the portals and the lung is reinflated with direct vision checking. Final C-arm fluoroscopy checking is performed to assess curvature correction.

If needed, the procedure is performed for TL/L curves via the retroperitoneal approach.

The day after surgery, weight-bearing is granted and full-spine standing X-rays are conducted. The thoracic drain is removed approximately after two days. The patient is discharged in about a week.

## 3. Results

### 3.1. Patients Demographics

The sample of the study consisted of 67 patients (91.04% female), aged 10 to 22 at the time of surgical procedure (mean 14.23 ± 2.26 years), who underwent AVBT with the modified technique as previously described. Mean follow-up time was 21.45 ± 7.11 months, ranging from 13.3 to 39.9 months. Length of stay in hospital ranged from 4 to 15 days postoperatively (mean 8.42 ± 2.26 days).

Skeletal maturity degree at the time of surgery was assessed using both Risser staging (2.94 ± 1.48, ranging from stage 0 to stage 5) and Sanders Maturity Scale staging (5.78 ± 1.55, ranging from stage 2 to stage 8).

### 3.2. Preoperative Diagnosis and Classification

All the patients were affected by adolescent idiopathic scoliosis (AIS). The main structural curve was right-convex in 83.58% of patients.

According to Lenke classification, there were 42 Type 1 (62.69%), 2 Type 2 (2.99%), 11 Type 3 (16.42%), 8 Type 5 (11.94%), 4 Type 6 (5.97%) curves. No Type 4 curves were included in this study. Lumbar spine modifier A was the most prevalent (29 patients, 43.28%), followed by lumbar modifier C (22 patients, 32.84%) and lumbar modifier B (13 patients, 23.88%). Sagittal thoracic alignment was neutral in 48 patients (71.64%), hypokyphotic in 12 patients (17.91%) and hyperkyphotic in 7 patients (10.45%).

### 3.3. Surgical Data

Of the 67 AVBT procedures performed, 48 (71.64%) were thoracic, 13 (19.40%) were thoracolumbar and 6 (8.96%) were double-sided (thoracic and thoracolumbar). Operative time ranged from 156 to 550 min (mean 266.94 ± 89.87 min).

A total of 16 surgical complications were recorded in 12 patients, with an overall surgical complication rate of 17.91%. Complications observed after surgery included persistent pneumothorax (*n* = 5, 7.46%), pleural effusion (*n* = 3, 4.48%), pneumonia (*n* = 3, 4.48%), upper limb peripheral neurapraxia due to surgical positioning (*n* = 2, 2.99%), hemothorax (*n* = 1, 1.49%), CSF hypotension (*n* = 1, 1.49%) and conversion to PSF (*n* = 1, 1.49%).

### 3.4. Axial Correction

Axial rotation was assessed by the Nash-Moe index at the apex vertebra of major structural curves. An average 25.74% de-rotation of structural main thoracic (MT) curves was reported postoperatively (from mean Nash-Moe value of 1.84 ± 0.71 to 1.36 ± 0.73) (*p* < 0.001), and 27.39% was reported at the most recent follow-up (from 1.84 ± 0.71 to 1.33 ± 0.69) (*p* < 0.001).

The mean Nash-Moe index of structural thoracolumbar/lumbar (TL/L) curves improved by 23.64% immediately after surgery (from 1.00 ± 1.00 to 0.76 ± 0.86) and by 21.21% at the final follow-up (from 1.00 ± 1.00 to 0.79 ± 0.89), but these findings were not statistically significant.

### 3.5. Coronal Correction

The extent of coronal deviation at follow-up decreased with statistical relevance in all curves included in this study.

The mean postoperative Cobb Angle of MT curves was 36.57 ± 10.65°. There was a 29.71% correction (mean preoperative width of 52.03 ± 13.04°), which was statistically significant (*p* << 0.001). This correction was maintained at the most recent follow-up (mean 39.50 ± 11.09°) (*p* << 0.001).

The average preoperative TL/L curves’ Cobb Angle was 38.79 ± 13.79°, which decreased to 26.40 ± 10.57° after surgery (*p* << 0.001) and 29.67 ± 10.45° at the latest follow-up (*p* < 0.001).

The PT curves showed a postoperative and final follow-up reduction of 73.14% (*p* < 0.001) and 16.14% (*p* < 0.028), respectively (from 24.93 ± 8.07° to 6.70 ± 5.65° and 20.91 ± 6.91°).

Among patients with Lenke type 1, 2 and 3 scoliosis, the MT curves had a correction of 29.85% (from mean preoperative width of 54.81 ± 11.86° to 38.45 ± 10.19°) postoperatively (*p* << 0.001) and of 24.11% at the most recent follow-up (38.45 ± 10.19°) (*p* << 0.001) was reported. TL/L curves correction of 26.93% after surgery (from 35.15 ± 11.83° to 25.69 ± 10.50°) (*p* << 0.001), and of 21.87% at the latest follow-up (27.47 ± 8.76°) (*p* < 0.003) was reported. Correction of the PT curves postoperatively and at final follow-up was 75.06% (*p* << 0.001) and 17.89% (*p* < 0.016), respectively (from 25.03 ± 8.14° to 6.24 ± 5.08° and 20.55 ± 6.79°).

The Cobb Angle values in patients with Lenke type 5 and 6 scoliosis demonstrated a postoperative reduction of 45.22% (mean preoperative width of 53.22 ± 8.92° to 29.16 ± 11.22°) (*p* << 0.001) and a final reduction of 25.63% (39.58 ± 12.45°) (*p* < 0.050).

The Average Clavicle Angle correction at follow-up reached 82.17% (from −2.29 ± 3.44° to −0.41 ± 2.55°) (*p* < 0.002).

Coronal Balance had no statistically significant difference between preoperative, postoperative and final follow-up.

Trunk Shift values decreased from 15.48 ± 19.04 mm to 7.88 ± 15.53 mm postoperatively (−49.08%, *p* < 0.024) and to 5.28 ± 14.99 mm (−65.90%, *p* < 0.008) at the most recent follow-up.

### 3.6. Sagittal Correction

There was no statistically significant difference between the mean Thoracic Kyphosis (TK) measured preoperatively (26.32 ± 12.99°), postoperatively (28.36 ± 14.26°) (*p* < 0.434) and at the final follow-up (29.77 ± 14.30°) (*p* < 0.260).

Lumbar Lordosis did not decrease significantly from a preoperative mean of 57.02 ± 11.76° to 53.17 ± 12.39° (*p* < 0.097) postoperatively and to 57.83 ± 11.20° (*p* < 0.749) at the most recent follow-up.

The mean preoperative Thoracic Height was 285.33 ± 31.72 mm, which increased to 298.63 ± 22.807 mm after surgery and further increased to 301.16 ± 29.98 mm (*p* < 0.022) at the latest follow-up (*p* < 0.013).

Trunk Height averaged 452.83 ± 48.12 mm preoperatively, 471.65 ± 28.87 mm postoperatively (*p* < 0.015) and 452.80 ± 42.70 mm (*p* < 0.998) at the final follow-up.

### 3.7. Tables and Figures

The results of this study are reported in Table 1 and Table 2.

The technical variant of AVBT is depicted in Figure 1.

## 4. Discussion

This study compares the three-dimensional spine parameters of a heterogeneous cohort of patients with adolescent idiopathic scoliosis (AIS), referred to the Regional Spinal Department of the University and Hospital Trust of Verona (Italy) and undergoing Anterior Vertebral Body Tethering (AVBT) correction surgery with a modified technique, as previously described, performed by the same senior spine surgeon between 2020 and 2024. Preoperative (Preop), postoperative (Postop) and latest follow-up (FU) clinical and imaging data were gathered (mean follow-up at 21.45 ± 7.11 months). Patients in this study’s cohort were aged between 10 and 22 (mean 14.23 ± 2.26 years) and had a bone maturity grade estimated by Risser radiographic staging, obtained from AP pelvic radiograms, between 0 and 5 (mean 2.94 ± 1.48). All the patients in this study were affected by adolescent idiopathic scoliosis of various grades according to Lenke classification on radiograms of the spine in AP and LL projection: grade 1 (n = 42), grade 2 (n = 2), grade 3 (n = 11), grade 4 (n = 0), grade 5 (n = 8) and grade 6 (n = 4). Thus, the patient population had differentiating features in terms of both scoliotic curve type and bone maturity. Despite the various curve patterns in this study’s group of patients, the number of AIS patients characterized by a thoracic-type major structured curve (Lenke 1, 2, 3) was higher than the number of those characterized by a lumbar-type major structured curve (Lenke 5 and 6). For this reason, the same statistical analyses applied to the entire patient cohort were also performed on the Lenke 1, 2, 3, 5, and 6 subgroups. The bone age of this population was also very heterogeneous, with Risser values varying between 0 and 5; although many previous studies examined patients characterized by a low degree of bone maturity, there is scientific evidence suggesting that the correction values of the structured and compensatory scoliotic curves measured in the sagittal and coronal plane in the immediate postoperative period are similar in patients with different degrees of bone maturity [7,8,9,10,11,12,13,14,15]. However, a good correction was obtained immediately after the modified surgical procedure in contrast to the late “Hueter-Volkmann” correction of the curves as reported in the literature. To increase curve reduction, we performed a hemi-discectomy on the convex side at the curve apex. This stops the growth of the apical intervertebral disc on the convex side.

### 4.1. Axial Correction

The extent of axial de-rotation of the apical vertebra in each major structural curve was assessed by Nash-Moe radiographic index on radiograms of the spine in AP projection obtained in preop, postop and FU conditions. Based on the data collected, a statistically significant improvement in vertebral rotation values of the apical vertebrae of the structural MT curves was observed, with an average improvement in the magnitude of rotation of 25.74% (*p* < 0.001) at the postoperative follow-up and an even greater de-rotation obtained at the most recent follow-up compared to preoperative values (27.39%, *p* < 0.001). Only a few studies have previously focused on evaluating the correction of vertebral rotation in the axial plane in AIS patients treated by AVBT. Furthermore, data were often collected indirectly by measuring the thoracic angle of trunk rotation. Many of these studies showed no statistically significant improvement in thoracic trunk rotation angle at the first postoperative follow-up compared with preoperative assessment [11,16]. Although the degree of rotational deformity was measured differently (Nash-Moe radiographic classification versus thoracic angle of trunk rotation), the results obtained by these authors appear to be in sharp contrast to those obtained of this study: it can be hypothesized that our results are attributable to the specific surgical approach adopted, which enables a slight reduction in the scoliosis and a partial de-rotation of the curves. Although there is currently no scientific evidence to suggest the possibility of correction of axial deformity in the period following AVBT treatment in accordance with the Hueter-Volkmann law, it is however possible to assume that similarly to coronal curve correction, there might be the possibility of further axial rotation correction following surgery, as observed in this study. Further future studies, especially conducted on patient populations with low skeletal maturity level, would be needed to confirm this hypothesis.

The efficacy of de-rotation in posterior spinal fusion (PSF) techniques has been established by substantial evidence. Kadoury et al. report 64% correction of axial deformity of apical vertebrae using the pedicle screw technique, 33% correction with the Cotrell-Dubousset technique, and approximately 74% correction with Suk’s Direct Vertebral Rotation (DVR) technique [17]. Araujo et al. reported a 32.14% correction of axial deformity of apical vertebrae estimated by Nash-Moe radiographic classification, using the pedicle screws technique in scoliotic curves with mean preoperative coronal Cobb values of 78.3° [18]. Although evidence suggests better control of vertebral rotation with PSF techniques, the technical variant of AVBT proposed in this paper may offer a viable surgical alternative to approach axial deformity of the spine.

### 4.2. Coronal Correction

The magnitude of coronal deformation in each scoliotic curve was determined by measuring the Cobb Angle on full spine standing radiographs in AP projection obtained in the preop, postop and FU conditions. Based on the data collected, a statistically significant improvement in the coronal Cobb Angle values was observed, with an average correction of the MT curves of 29.71% (*p* << 0.001) obtained immediately after surgery and of 24.08% at the latest follow-up as opposed to preoperative values. The TL/L curves’ deformity reduced by 31.95% following surgery (*p* << 0.001) and by 21.87% at the final follow-up (*p* < 0.001), in comparison with preop assessments. In particular, the average correction of PT and MT curves was even higher in the cohort including only AIS patients characterized by a structured major thoracic curve (Lenke 1, 2 and 3 subgroup), with an average postop improvement of 75.06% of the PT curves (*p* << 0.001) and of 29.85% of the MT curves (*p* << 0.001). Many authors evaluated the efficacy of coronal correction with the AVBT technique; they obtained similar data to those reported in this study. In a systematic review by Zhu et al., which included 1045 patients of 26 studies, a mean MT curve correction of about 46.6% ± 13.8% was reported [19]. These results are in line with our retrospective trial. However, the effectiveness of PSF techniques is always greater than that of AVBT in correcting coronal deformity. According to the findings of Kadoury et al., PSF techniques have shown equal effectiveness in correcting coronal deformity [17]. Shin et al. found residual coronal curves in the immediate postoperative time in the cohort of patients treated by AVBT to be superimposable to those of the patients operated by PSF but starting from different mean Cobb values (AVBT: 46.0°; PSF: 55.3°) [16]. Newton et al., comparing two cohorts of 23 AVBT and 26 PSF patients, showed a lower coronal correction in the AVBT group (43%) than in the PSF one (69%) [11]. Lonner et al. showed a coronal correction rate of MT curves treated by PSF of about 65% ± 11% [3]. Vavruch et al. showed a coronal MT curve correction, in PSF-treated patients, of about 73% ± 12% [20]. As far as it is known, posterior stabilization techniques enable greater dominance of coronal and axial deformity planes than AVBT. However, in a selected group of patients, AVBT avoids the invasive vertebral stabilization of PSF techniques that limits the range of motion of the whole spine, with a great reduction in the quality of life. AVBT also does not preclude, in case of failure, definitive posterior arthrodesis.

### 4.3. Sagittal Correction

The entity of sagittal deformity in each scoliotic curve was calculated by measuring the Cobb Angle on spine radiograms in the LL projection obtained in preop, postop and FU conditions. Based on the data collected and in line with what has been previously reported by other authors in the medical literature, no statistically significant sagittal parameter modifications were shown in this trial [7,10,11,13,15]. This finding suggests that, in contrast to PSF techniques such as the Cotrell-Dubousset technique and the pedicle screw stabilization technique that enable correction of deformities in the sagittal plane and are often associated with reduced Thoracic Kyphosis, AVBT does not affect sagittal balance.

### 4.4. Surgical Complications

This study’s overall complication rate was 17.91%. Pulmonary complications were the most frequently observed: five patients developed persistent pneumothorax after surgery that required thoracic drain to be maintained for a longer time, associated with pulmonary rehabilitation; three patients developed self-limiting pleural effusion; and three patients developed pneumonia that resolved after antibiotic treatment. One patient required an unplanned conversion to PSF for insufficient correction achieved after AVBT.

Shin et al., in their systematic review of outcome studies of AVBT, reported an average complication rate of 26% [16]. Roser et al.’s reported complication rate was 23% [21]. The complication rate of the technical variant of AVBT proposed in this study seems comparable to that of the standard AVBT technique described in the literature.

## 5. Conclusions

Our retrospective observational trial reported that the AVBT technique, in combination with hemi-discectomy at the apex of the curve and a proximal–distal tightening of the PET tether, can achieve immediate correction of scoliosis deformity not only in the coronal plane but also in the axial plane, thus overcoming the traditional Hueter-Volkmann principle, with a mean operating time and a surgical complication rate comparable to the standard AVBT technique. These results suggest that the proposed AVBT technical variant may be a viable surgical alternative to the gold-standard PSF techniques if appropriate indication criteria are met and the surgical procedure is performed by an expert spine surgeon. However, to confirm these findings, further high-quality studies with a larger sample size and longer-term follow-up are required.

## Figures and Tables

**Table 1:** Patient demographics, curve classification and surgical data.

Demographics			
Number of patients	67	Follow-up (months) †	21.45 ± 7.11 (range 13.3 to 39.9)
Age (years) †	14.23 ± 2.26 (range 10 to 22)	Length of stay in hospital (days) †	8.42 ± 2.26 (range 4 to 15)
Sex *		Sanders Maturity Scale stage *	
Male	6 (8.96%)	mean	5.78 ± 1.55
Female	61 (91.04%)	1	0 (0%)
Risser skeletal maturity stage *		1	2
mean	2.94 ± 1.48	3	2 (2.99%)
0	4 (5.97%)	4	8 (11.94%)
1	10 (14.93%)	5	13 (19.40%)
2	10 (14.93%)	6	14 (20.90%)
3	14 (20.90%)	7	25 (37.31%)
4	20 (29.85%)	8	3 (4.48%)
5	9 (13.43%)	8	3 (4.48%)
**Preoperative Lenke Classification**			
Major curve convexity *			
Right	56 (83.58%)		
Left	11 (16.42%)		
Lenke type curve *		Lumbar spine modifier *	
1	42 (62.69%)	A	29 (43.28%)
2	2 (2.99%)	B	16 (23.88%)
3	11 (16.42%)	C	22 (32.84%)
4	0 (0%)	Sagittal thoracic alignment *	
5	8 (11.94%)	Hypokyphotic	12 (17.91%)
6	4 (5.97%)	Neutral	48 (71.64%)
		Hyperkyphotic	7 (10.45%)
**Surgical Data**			
Surgical Procedure Type		Mean operative time (minutes) †	266.94 ± 89.87 (range 156 to 550)
Thoracic AVBT *	48 (71.64%)		
Thoracolumbar AVBT *	13 (19.40%)		
Double-sided Thoracic and Thoracolumbar AVBT *	6 (8.96%)		
Complication rate	17.91% (12/67 patients)		
Persistent pneumothorax	5 (7.46%)	Hemothorax	1 (1.49%)
Pleural effusion	3 (4.48%)	CSF hypotension	1 (1.49%)
Pneumonia	3 (4.48%)	Upper Limb peripheral neuropraxia	2 (2.99%)
Conversion to PSF	1 (1.49%)		

* Values are given as the count, with percentage in parentheses; † Values are given as the mean and standard deviation; AVBT: Anterior Vertebral Body Tethering.

**Table 2:** Comparison between preoperative, postoperative and final follow-up radiographical values.

	Preop	PostOp		Final F.U.		Mean Correction	
						P.O.	Final F.U.
**Axial correction**							
Nash-Moe index *							
Major structural curves							
MT	1.84 ± 0.71	1.36 ± 0.73	*p* < 0.001	1.33 ± 0.69	*p* < 0.002	25.74%	27.39%
TL/L	1.00 ± 1.00	0.76 ± 0.86	*p* = 0.183	0.79 ± 0.89	*p* = 0.302	23.64%	21.21%
**Coronal correction**							
Cobb Angle (°) *							
All curves							
MT	52.03 ± 13.04	36.57 ± 10.65	*p* << 0.001	39.50 ± 11.09	*p* << 0.001	29.71%	24.08%
PT	24.93 ± 8.07	6.70 ± 5.65	*p* << 0.001	20.91 ± 6.91	*p* = 0.028	73.14%	16.14%
TL/L	38.79 ± 13.79	26.40 ± 10.57	*p* << 0.001	29.67 ± 10.45	*p* < 0.001	31.95%	23.51%
Lenke 1, 2 and 3 curves							
MT	54.81 ± 11.86	38.45 ± 10.19	*p* << 0.001	38.45 ± 10.19	*p* << 0.001	29.85%	24.11%
PT	25.03 ± 8.14	6.24 ± 5.08	*p* << 0.001	20.55 ± 6.79	*p* < 0.016	75.06%	17.89%
TL/L	35.15 ± 11.83	25.69 ± 10.50	*p* << 0.001	27.47 ± 8.76	*p* < 0.003	26.93%	21.87%
Lenke 5 and Lenke 6 curves							
TL/L	53.22 ± 8.92	29.16 ± 11.22	*p* << 0.001	39.58 ± 12.45	*p* = 0.050	45.22%	25.63%
Clavicle Angle (°) *	−2.29 ± 3.44	−0.41 ± 2.55	*p* < 0.002	−1.65 ± 2.29	*p* = 0.293	82.17%	27.98%
Coronal Balance (mm) *	−1.42 ± 17.59	0.07 ± 18.87	*p* = 0.666	−6.51 ± 16.91	*p* = 0.181	104.85%	−356.92%
Trunk Shift (mm) *	15.48 ± 19.04	7.88 ± 15.53	*p* = 0.024	5.28 ± 14.99	*p* < 0.008	49.08%	65.90%
**Sagittal correction**							
Thoracic Kyphosis (°) *	26.32 ± 12.99	28.36 ± 14.26	*p* = 0.434	29.77 ± 14.30	*p* = 0.260	−7.75%	−13.11%
Sagittal Balance (mm) *	−9.44 ± 31.69	10.01 ± 28.47	*p* < 0.001	−4.82 ± 29.63	*p* = 0.492	206.02%	48.93%
Lumbar Lordosis (°) *	57.02 ± 11.76	53.17 ± 12.39	*p* = 0.097	57.83 ± 11.20	*p* = 0.749	6.75%	−1.41%
Thoracic Height (mm) *	285.33 ± 31.72	298.63 ± 22.80	*p* < 0.013	301.16 ± 29.98	*p* = 0.022	−4.66%	−5.55%
Trunk Height (mm) *	452.83 ± 48.12	471.65 ± 28.87	*p* < 0.015	452.80 ± 42.70	*p* = 0.998	−4.16%	0.01%

* Values are given as the mean and standard deviation; MT: main thoracic curve; PT: proximal thoracic curve; TL/L: thoracolumbar–lumbar curve; P.O.: postoperative; F.U.: follow-up.

## Data Availability

Data are contained within the article.
